# In vitro assessment of the antimicrobial activity of wound dressings: influence of the test method selected and impact of the pH

**DOI:** 10.1007/s10856-014-5343-9

**Published:** 2015-01-13

**Authors:** Cornelia Wiegand, Martin Abel, Peter Ruth, Peter Elsner, Uta-Christina Hipler

**Affiliations:** 1 Department of Dermatology, University Hospital Center Jena, Erfurter Str. 35, 07740 Jena, Germany; 2 Lohmann & Rauscher GmbH & Co. KG, Westerwaldstraße 4, 56579 Rengsdorf, Germany

## Abstract

Antibacterial activity of dressings containing antimicrobials is mostly evaluated using in vitro tests. However, the various methods available differ significantly in their properties and results obtained are influenced by the method selected, micro-organisms used, and extraction method, the degree of solubility or the diffusability of the test-compounds. Here, results on antimicrobial activity of silver-containing dressings obtained by agar diffusion test (ADT), challenge tests (JIS L 1902, AATCC 100), and extraction-based methods (microplate laser nephelometry (MLN), luminescent quantification of bacterial ATP (LQbATP)) using *Staphylococcus aureus* and *Pseudomonas aeruginosa* were evaluated. Furthermore, the effect of the pH on antibacterial efficacy of these dressings was investigated. All silver-containing dressings exerted antimicrobial activity in all in vitro tests and results correlated considerably well. Differences were observed testing the agent-free basic materials. They did not exhibit any antimicrobial effects in the ADT, MLN or LQbATP, since these methods depend on diffusion/extraction of an active agent. However, they showed a strong antimicrobial effect in the challenge tests as they possess a high absorptive capacity, and are able to bind and sequester micro-organisms present. Therefore, it seems recommendable to choose several tests to distinguish whether a material conveys an active effect or a passive mechanism. In addition, it could be shown that release of silver and its antimicrobial efficacy is partially pH-dependent, and that dressings themselves affect the pH. It can further be speculated that dressings’ effects on pH and release of silver ions act synergistically for antimicrobial efficacy.

## Introduction

Treatment of wound infections has an important role in wound management. A high bioburden will interrupt the normal wound healing process and may lead to the formation of persistent, non-healing wounds [1–3]. Hence, dressings containing antimicrobial substances, most commonly silver, are increasingly utilized. Silver (Ag^+^) is effective against a broad range of micro-organisms such as yeast, mold and bacteria, including MRSA and VRE, when it is provided in appropriate concentration [1, 4, 5].

The antibacterial activity of these dressings is mostly evaluated using in vitro tests such as the agar diffusion test (ADT), challenge tests like the JIS L 1902 or the AATCC 100, as well as new methods such as microplate laser nephelometry (MLN) and luminometric quantification of bacterial ATP (LQbATP). These tests allow a direct comparison of the effects of the dressings on the micro-organisms. Ideally, they are simple, rapid, reproducible, inexpensive, and enable handling of a range of sample quantities [6]. However, the various test methods available differ significantly in their properties and hence in their outcome. The results obtained are influenced by the method selected and the microorganisms used as well as by the extraction method or the degree of solubility or diffusability of each test-compound [6].

The agar diffusion test (ADT) can be utilized to measure the effect of an antimicrobial sample against micro-organisms grown on culture plates [7, 8]. Therefore, microorganisms are swabbed uniformly across a culture plate and the test sample is applied onto the agar. The antimicrobial agent then has to diffuse from its point of application into the agar. The concentration of the agent will be highest next to the application point, and will decrease with increasing distance. If the agent is effective against the microorganisms at a certain concentration, no colonies will grow where the concentration in the agar is greater than or equal to the effective concentration. This is the zone of inhibition (ZOI). Accordingly, the size of the ZOI can be used to rate the agent’s antimicrobial efficacy: the larger the ZOI, the more effective the agent. This method further strictly depends on the diffusion of the antimicrobial agent in the wound dressing. Large or highly charged molecules, e.g. PHMB, might exhibit lower diffusion capacities compared to small ions such as Ag^+^. Moreover, permanently bound agents cannot diffuse from the sample into the agar and might not elicit a result in the ADT while showing a high antimicrobial activity in challenge tests.

Challenge tests, such as the JIS L 1902 or AATCC 100, analyse the antimicrobial efficacy of the material after direct contact with the microorganisms over a respective time period. In contrast to the ADT, they are independent from the diffusion properties of the antimicrobial agent. In addition, they allow a quantitative evaluation of antimicrobial activity as results are retrieved as percentage reduction of microbial counts [9] or inhibition of microbial growth in log-scale [10]. However, these test methods are labour-intensive and time-consuming resulting in difficulties if large sample numbers have to be processed.

High-throughput screening approaches for determining the reaction of microorganisms to antimicrobial agents mostly relay on the measurement of the cellular adenosine triphosphate (ATP) content or observation of microbial growth by determination of turbidity. As ATP is found in all living and metabolic active cells, it can be used to determine the amount of viable microbial cells present. It can be quantified using a bioluminescence assay comprised of the enzyme luciferase from Photinus pyralis and d-luciferin, the enzyme’s substrate. Luciferin is converted into oxyluciferin in an ATP-, Mg^2+^- and oxygen dependent reaction which generates a yellow–green luminescent light signal [11, 12]. The amount of emitted light is directly proportional to the ATP content [11, 13], and hence, a linear function of the number of living cells in the suspension [14].

However, the turbidity of the respective medium itself can be used to monitor the growth of microorganisms. While turbidimetry obeys Beer’s Law and requires relatively high concentrations of particles to be measured, nephelometry is a direct method to measure light scattered already by relatively low amounts of particles in solution at right or forward angle to a laser beam. The most common application of laser-based nephelometry in microplate format, designated as microplate laser nephelometry (MLN) is the fully automated solubility screen in HTS laboratories [15]. Nephelometry is further used in clinical chemistry to determine serum immunoglobulin (IgA, IgG, IgM), complement components (C3, C4), acute phase reactant proteins (CRP, transferring), albumin, and α-1-antitrypsin by protein precipitation or in organic chemistry to quantify macromolecules, e.g. monitoring of a polymerisation reaction. MLN presents a valuable tool to investigate the effect of antimicrobial substances on the growth of microorganisms, as it allows high-throughput screening, incubation over a prolonged time period, and in situ-monitoring of changes in the dose–response curves as well as the IC_50_, the half maximum inhibitory concentrations [16–19]. Both, MLN and LQbATP are performed in solution and wound dressing extracts were prepared according to DIN EN ISO 10993-12 as previously reported [20, 21].

The presented study describes the comparison of the results of antimicrobial activity of silver-containing dressings obtained by ADT, JIS L 1902 and AATCC 100, as well as MLN and LQbATP using *Staphylococcus aureus* and *Pseudomonas aeruginosa*, which are the most prominent bacteria in wound infection [22, 23], as model organisms.

Moreover, as only limited knowledge exists concerning the effect of the wound pH on the antibacterial efficacy of antimicrobial-containing dressings, ADT and MLN were elected to investigate the influence of pH on antimicrobial activity of silver-containing dressings. Chronic wounds most commonly have a pH range of 6.5–8.5 [24, 25]. This shift towards higher pH values in chronic wounds compared to acute wounds is called ‘alkaline shift’ and is thought to be due to both, tissue necrosis and the presence of microorganisms. Recent studies relating to the influence of the pH on the performance of antiseptics revealed that bactericidal activity of chlorhexidine and octenidine was mainly pH-independent in pH range from 5.0 to 9.0 while a most pronounced influence was observed for polihexanide and PVP-iodine (own unpublished results). In addition, in vitro studies have shown that wound dressings can have significant effects on the pH [26]. Hence, it is of interest to further determine the effect of the dressing itself on the surrounding pH as the establishment of a low physiological pH might also aid wound healing.

## Materials and methods

### Materials

The following wound dressings were selected for this study: alginate (Suprasorb^®^ A, Lohmann & Rauscher GmbH & Co. KG, Germany) and alginate + ionic-Ag (Suprasorb^®^ A + Ag, Lohmann & Rauscher GmbH & Co. KG, Germany), alginate + nano-Ag (Acticoat^◊^ Absorbent, Smith & Nephew GmbH, Germany), sodium carboxymethylcellulose (CMC) (AQUACEL^®^, ConvaTec GmbH, Germany) CMC with Ag^+^ (AQUACEL^®^ Ag, ConvaTec GmbH, Germany) as well as polyurethane (PU)-foam with TLC (UrgoCell^®^, URGO GmbH, Germany) and PU-foam with TLC/Ag^+^ (UrgoCell^®^ silver, URGO GmbH, Germany). Active ingredients and wound dressing basic materials are specified in Table 2 according to the manufacturers’ descriptions.

*Staphylococcus aureus* ATCC 6538 and *P. aeruginosa* ATCC 27853 were obtained from the DSMZ (Deutsche Sammlung von Mikroorganismen und Zellkulturen, Germany). For cultivation of bacteria, special peptone and “lab-lemco” powder for preparation of caso-bouillon and bacteriological agar were purchased from Oxoid (UK). Columbia agar plates with 5 % sheep blood and MH2 agar plates were acquired from Biomeriéux (France).

0.9 % NaCl solution was purchased from Fresenius Kabi Deutschland GmbH (Germany). 1 N HCl, 1 N NaOH, and Tween 20 were obtained from Carl Roth GmbH (Germany).

### Culture of* S. aureus* and * P. aeruginosa*

Seed stocks of *S. aureus* and *P. aeruginosa* were kept on Columbia agar plates. For experiments, overnight cultures were prepared by inoculation of 20 mL caso-bouillon (pH 7.0) with 1–2 colonies of the respective test organism. Overnight cultures were incubated for 16 h at 37 °C under shaking.

### Evaluation of antibacterial activity by ADT

The agar diffusion test (ADT) was performed in accordance with the DIN 58940-3. *Staphylococcus aureus* and *P. aeruginosa* overnight cultures were diluted 1:100 to adjust the working suspensions to a microbial count of app. 1–5 × 10^6^ cfu/mL. 100 µL of these suspensions were plated on MH2-agar plates and left to dry for 10 min. Afterwards aseptically prepared dressing samples (with a diameter of 6 mm) were placed onto the inoculated agar plates along with the negative control (additive-free bio-discs; Biomeriéux, France) and the respective positive control (*S. aureus*: bio-discs with 30 µg vancomycin, *P. aeruginosa*: bio-discs with 10 µg gentamycin; Biomeriéux, France). All samples and controls were wetted with 20 µL of 0.9 % NaCl. The plates were incubated for 24 h at 37 °C. Afterwards the zone of inhibition (ZOI) was measured in mm and all plates were photographed for documentation.

### Assessment of the antibacterial activity according to JIS L 1902

Testing for antibacterial activity was carried out in accordance to the Japanese industrial standard (JIS L 1902:2002, “Testing method for antibacterial activity of textiles”) as reported previously [20]. In brief, overnight cultures of *S. aureus* and *P. aeruginosa* were diluted 1:1,000 to adjust a microbial count of app. 1–2 × 10^5^ cfu/mL. For experiments, 400 mg samples of the wound dressings were aseptically prepared and inoculated with 200 µL test microbe solution and incubated for 24 h at 37 °C under aerobic conditions. Polyester material (I.T.S. Textilhandels GmbH, Austria) was used as growth control. For determination of the germ number, the incubated samples were extracted in 0.9 % NaCl solution supplemented with 0.2 % Tween 20. Serial dilutions were plated onto Columbia agar plates and incubated for 24 h at 37 °C. Subsequently, colonies were counted, total cfu (colony forming units) determined, and growth reduction calculated according to Eq. (1). A logarithmic microbial growth reduction of less than 0.5 represents no antibacterial activity. Values between 0.5 and 1 are rated as a slight, values greater than 1 and less or equal to 3 as a significant, and a log reduction greater than 3 as a strong antibacterial activity.

1 $${\text{log growth reduction}}_{{( 2 4 {\text{ h}})}} = {\text{ log cfu}}\left( {\text{negative control}} \right)_{{( 2 4 {\text{ h}})}} {-}{\text{ log cfu}}\left( {\text{sample}} \right)_{{( 2 4 {\text{ h}})}}$$

### Measurement of the antibacterial efficacy according to AATCC 100

Antibacterial efficacy was determined according to the AATCC test method 100 (AATCC 100-2004, “Assessment of antibacterial finishes on textile materials”). *Staphylococcus aureus* and *P. aeruginosa* overnight cultures were diluted 1:1,000 to adjust a microbial count of app. 1–2 × 10^5^ cfu/mL. For experiments, circular swatches (d = 5 cm) of the wound dressings were aseptically prepared and incubated with 1 mL test microbe solution for 24 h at 37 °C under aerobic conditions. Polyester material (I.T.S. Textilhandels GmbH, Austria) was used as growth control. After incububation, samples are transferred to a neutralizing solution (0.9 % NaCl solution with 0.2 % Tween 20). For determination of the germ number, serial dilutions were plated onto Columbia agar plates and incubated for 24 h at 37 °C. Subsequently, colonies were counted, total cfu (colony forming units) determined, and growth reduction calculated according to Eq. (2). A germ reduction of less than 50 % represents no antibacterial efficacy. Values between 50 and 90 % are rated as a significant and a germ reduction greater than 90 % as a strong antibacterial efficacy. Furthermore, to allow comparison between AATCC 100 and JIS L 1902, the results retrieved were also subjected to calculation of antibacterial activity according to Eq. (1).

2 $${\text{Germ reduction [\% ] = }}\frac{{ ( {\text{cfu(control)}}_{{ ( 0 {\text{ h)}}}} {\text{ - cfu(sample)}}_{{ ( 2 4 {\text{ h)}}}} )}}{{{\text{cfu(control)}}_{{ ( 0 {\text{ h)}}}} }}\; \times \; 1 0 0$$

### Determination of the antibacterial effect of wound dressings by MLN and LQbATP

MLN and LQbATP are solution-based methods, hence, wound dressing extracts were prepared according to the standard used for evaluation of textile cytotoxicity (DIN EN ISO 10993-12) as previously reported [20, 21]. Briefly, 1 g of each aseptically treated wound dressing was incubated in 50 mL caso-bouillon in Erlenmeyer flasks (Greiner, Germany) at 37 °C for 24 h under shaking (ThermoBath, GFL, Germany). Afterwards, each wound dressing extract was filtered over gauze by centrifugation at 1,000 rpm to remove any insoluble material residues. This filtrate was then sterilized by passage through a 0.2 µm filter and distinguished as original extract (100 %).

Microplate laser nephelometry (MLN) was performed in accordance with NCCLS M27-A2 and DIN EN 27027 as reported previously [14, 16, 17]. In brief, 100 µL of each extract were put in triplicate into the respective wells of a sterile, clear 96-well microplate (Greiner bio-one, Germany). Blanks for each extract tested were run at every assay. 100 µL of *S. aureus* or *P. aeruginosa* cell suspension (overnight cultures diluted to a microbial count of app. 5 × 10^3^ cfu/mL) were put in the respective wells of the 96-well microplate containing the wound dressing extracts. Microplates were covered with a clear adhesive film (Greiner bio-one, Germany). The adhesive film was punctured with a 25-gauge needle at the right brim of the well to allow gas exchange. Microplates were then placed in the microplate lasernephelometer (NEPHELOstar Galaxy, BMG LABTECH, Germany) and incubated for 24 h at 37 °C. During incubation, microplates were shaken in the instrument except for the duration of the hourly measurement.

3 $${\text{Microbial growth }}\left( {\text{MLN}} \right) \, \left[ \% \right]_{{}} = \frac{{\sum {[{\text{RNU}}} ]_{{1 - 24\;{\text{h}}}} ( {\text{sample}})}}{{\sum {[{\text{RNU}}} ]_{{1 - 24\;{\text{h}}}} \left( {\text{control}} \right)}} \times 100$$

The effect of the wound dressing extracts on microbial viability was further determined by luminometric quantification of the bacterial ATP content (LQbATP) [14, 16] using the BacTiter-Glo™ Assay (Promega, Mannheim, Germany), which is based on the detection of light generated by the ATP dependent enzymatic conversion of d-luciferin to oxyluciferin by firefly luciferase. After MLN measurements, microplates were equilibrated at room temperature under agitation. 10 µL of the contents of each well were transferred into a white 96-well microplate (NUNC MaxiSorp™; Thermo Fisher Scientific, Germany) and mixed with 90 µL caso-bouillon. Afterwards, 100 µL BacTiter-Glo™ Reagent was added to each well. The microplates were further incubated under agitation for 5 min. Thereafter, luminescence was recorded using the LUMIstar Galaxy (BMG LABTECH, Germany). The ATP concentration per well was calculated using an ATP standard curve.

4 $${\text{Microbial growth }}\left( {\text{LQbATP}} \right) \, \left[ \% \right] = \frac{{{\text{ATP[nM]}}_{{ 2 4\;{\text{h}}}} ( {\text{sample)}}}}{{{\text{ATP[nM]}}_{{ 2 4\;{\text{h}}}} ( {\text{control)}}}} \,\times 100$$

### Evaluation of the pH effect on the antibacterial activity of silver dressings’ efficacy using ADT and MLN

The agar diffusion test (ADT) was carried out as described in *Evaluation of antibacterial activity by ADT* with the difference that instead of MH2 agar plates, caso agar plates with specific pH were employed. Caso-bouillon (pH 7.0) with different pH was prepared by addition of HCl yielding caso-bouillon with pH 6.0 and 5.0, and by adding NaOH producing pH 8.0 and 9.0, respectively. These media were supplemented with 1.5 % agar, carefully heated until the agar was dissolved, and then cast into Petri dishes (Greiner bio-one, Germany), 10 mL per dish, under sterile conditions. The prepared caso-agar plates were kept at 4 °C until use.

Microplate laser nephelometry (MLN) was executed according to the section *Determination of the antibacterial effect of wound dressings by MLN and LQbATP* using caso-bouillon with different pH. Caso-bouillon (pH 7.0) with specific pH was prepared by addition of HCl yielding caso-bouillon with pH 6.0, and by adding NaOH producing pH 8.0 and 9.0, respectively. These media were used in the extraction step as well as for preparation of the *S. aureus* or *P. aeruginosa* cell suspensions with a microbial count of app. 5 × 10^3^ cfu/mL. The half maximal inhibitory concentration (IC_50_) of the dressing extracts under the experimental conditions used was calculated from the growth curves over 24 h. The ‘area under the curve’ was determined from the results for each antiseptic concentration tested and calculated as percentage of the untreated control. This was used to realize a dose–response curve for each antiseptic tested, from which the IC_50_ was calculated using a logistic fit function (y = A2 + (A1−A2)/(1 + (x/x0)^p); A1: upper limit, A2: lower limit, x0: IC_50_, p: slope of the curve; Origin 7.5, OriginLab, U.S.).

### Statistical analysis

One-way analysis of variance was carried out to determine statistical significances (Microsoft^®^ Excel 2000). Differences were considered statistically significant at a level of *P* < 0.05. Asterisks indicate significant deviations from the control at the respective incubation time (*P < 0.05; **P < 0.01; ***P < 0.001).

## Results

### Silver containing dressings exhibit a comparable antibacterial activity in the ADT

**Fig. 1:**
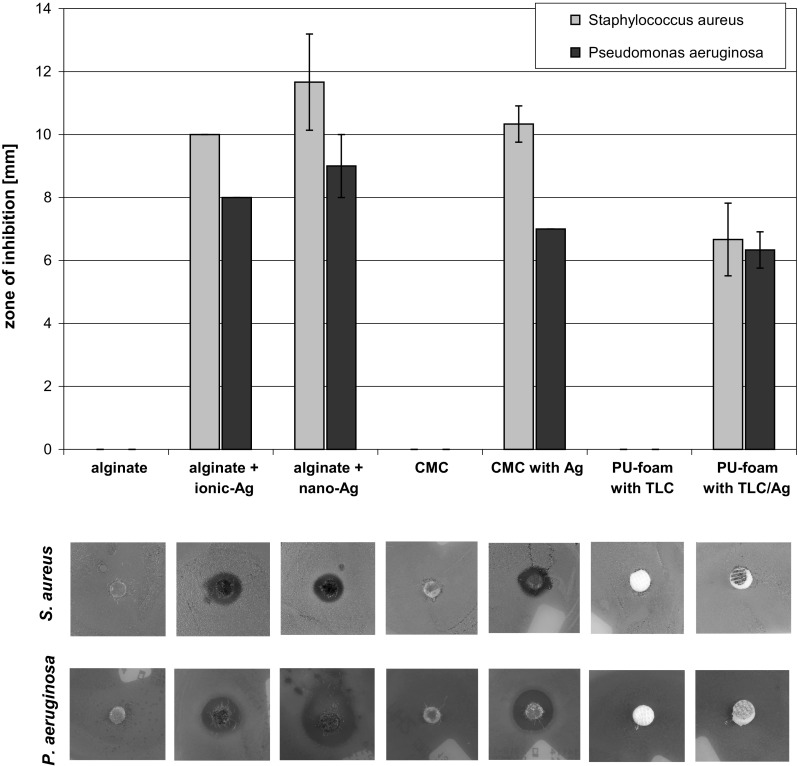
For the ADT, MH2 agar plates were inoculated with *S. aureus* or *P. aeruginosa* and incubated with the wound dressing samples at 37 °C for 24 h. Afterwards, the zone of inhibition (ZOI) was measured. Pictures show the photographic documentation of representative ADT results

Dressings without active agent, such as alginate, CMC and PU-foam with TLC, had no effect in the ADT (Fig. 1). On the other hand, the silver-containing dressings, alginate + ionic-Ag, alginate + nano-Ag and CMC with Ag^+^, exhibited a formation of a distinct zone of inhibition (ZOI) for both, *S. aureus* and *P. aeruginosa*. It was further noted that the effect on *S. aureus* was slightly higher compared to *P. aeruginosa*. In contrast, for PU-foam with TLC/Ag^+^ inhibition of bacterial growth was only found directly under the sample and no ZOI was formed.

### Reduction of microbial growth in the challenge tests JIS L 1902 and AATCC 100

A high effectiveness of both, agent-free and silver-containing dressings was observed in the challenge tests (Table 1). According to JIS L 1902, alginate, alginate + ionic-Ag, alginate + nano-Ag, CMC, CMC with Ag^+^ and PU-foam with TLC/Ag^+^ possess a strong antibacterial activity against *S. aureus*. Furthermore, a slight effect of PU-foam with TLC could also be observed. All dressings demonstrated a distinctly higher effect on *P. aeruginosa* compared to *S. aureus* and achieved a higher log reduction of the gram-negative bacteria compared to the gram-positive bacteria. Similar results were obtained in the AATCC 100 test (Table 2), All dressings showed a strong antibacterial efficacy against both, *S. aureus* and *P. aeruginosa*, with a growth reduction larger 90 %.

**Table 1:** Testing of antibacterial activity according to JIS L 1902 as log-reduction of microbial growth and according to AATCC 100 as decrease of viable germs in [%] revealed a strong antimicrobial effect for alginate, alginate + ionic-Ag, and alginate + nano-Ag against both *S. aureus* and *P. aeruginosa* as well as for CMC and CMC with Ag^+^

	JIS L 1902		AATCC 100			
	Microbial growth reduction [log cfu]		Germ reduction [%]		Microbial growth reduction [log cfu] (according to JIS L 1902)	
	*S. aureus*	*P.aeruginosa*	*S. aureus*	*P.aeruginosa*	*S. aureus*	*P.aeruginosa*
Control	0 ± 0.2	0 ± 0.2	0 ± 1.0	0 ± 1.0	0 ± 0.2	0 ± 0.2
Alginate	7.0 ± 0	7.0 ± 0	100 ± 0	100 ± 0	6.4 ± 0	8.9 ± 0
Alginate + ionic-Ag	7.0 ± 0	7.0 ± 0	100 ± 0	100 ± 0	6.4 ± 0	8.9 ± 0
Alginate + nano-Ag	7.0 ± 0	7.0 ± 0	100 ± 0	100 ± 0	6.4 ± 0	8.9 ± 0
CMC	4.0 ± 0.2	4.0 ± 0.2	100 ± 0	100 ± 0	6.4 ± 0	8.9 ± 0
CMC with Ag^+^	4.0 ± 0.7	4.0 ± 0.7	100 ± 0	100 ± 0	6.4 ± 0	8.9 ± 0
PU-foam with TLC	0.7 ± 0.2	0.7 ± 0.2	99.8 ± 0.1	0 ± 0.9	4.8 ± 1.7	2.2 ± 0.3
PU-foam with TLC/Ag^+^	3.3 ± 0.5	3.3 ± 0.5	100 ± 0	100 ± 0	6.4 ± 0	8.9 ± 0

PU-foam with TLC already had a slight antibacterial effect that was significantly increased in the silver-containing product PU-foam with TLC/Ag^+^. For comparison, the results of the AATCC 100 have also been evaluated according to the JIS L 1902 to yield log-reduction of microbial growth

**Table 2:** Wound dressing extracts were prepared in caso-bouillon with pH 6.0, 7.0, 8.0, or 9.0 by incubation for 24 h at 37 °C

Wound dressing	Basic material	Active ingredient	Effect on actual pH value at pH			
			6.0	7.0	8.0	9.0
Alginate + ionic-Ag	Alginate	Ionic silver	5.7	6.5	7.2	7.7
Alginate + nano-Ag	Alginate	Nano-crystalline silver	7.6	7.9	8.2	8.4
CMC with Ag^+^	Sodium carboxymethyl cellulose (CMC)	Ag^+^	5.6	6.1	7.1	8.1
PU-foam with TLC/Ag^+^	Polyurethane (PU) foam with TLC (lipidocolloid matrix)	Ag^+^	6.2	7.2	7.8	8.6

Afterwards, the actual pH was determined

For comparison of the results from the JIS L 1902 and the AATCC 100, the data from the latter was evaluated according to the log-reduction suggested by the JIS L 1902 (Table 1). The AATCC 100 differs from the JIS L 1902 in the way that more test material is used. Hence, mostly a higher effectiveness of the dressings was found in the AATCC 100 compared to the JIS L 1902 in the case of *S. aureus* as well as for *P. aeruginosa*.

### Determination of antibacterial activity using MLN and LQbATP

**Fig. 2:**
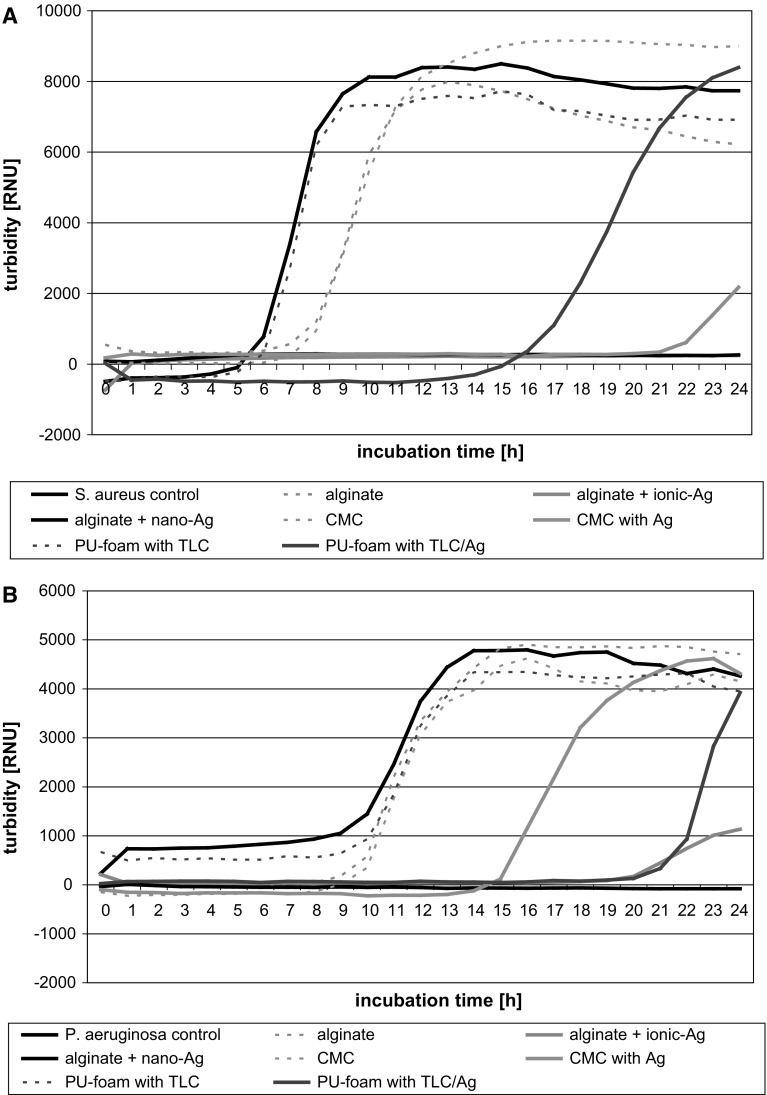
Growth curves of *S. aureus* (**a**) and *P. aeruginosa* (**b**) under the influence of the dressing extracts determined by MLN. It was found that extracts of silver-free dressings did not affect the growth of *S. aureus* and *P. aeruginosa* in solution. Extracts of silver-containing alginates effectively diminished bacterial growth, while CMC with Ag^+^ and PU-foam with TLC/Ag^+^ were only able to inhibit *S. aureus* and *P. aeruginosa* progeny

Microplate laser nephelometry (MLN) was used to monitor the growth of *S. aureus* (Fig. 2a) and *P. aeruginosa* (Fig. 2b) under the influence of the dressing extracts. In accordance to the ADT, a bactericidal effect was only observed in the case of the silver-containing dressings. The antibacterial activity of the extracts further depended on the extractability of the silver in the dressings. A complete inhibition of *S. aureus* and *P. aeruginosa* growth was achieved by extracts of alginate + ionic-Ag and alginate + nano-Ag. The extract of CMC with Ag^+^ demonstrated a significant reduction of *S. aureus* growth; however, silver concentrations reached were not high enough to abolish *P. aeruginosa* progeny. In contrast, extract of PU-foam with TLC/Ag^+^ accomplished a higher effect against *P. aeruginosa* compared to *S. aureus*.

**Fig. 3:**
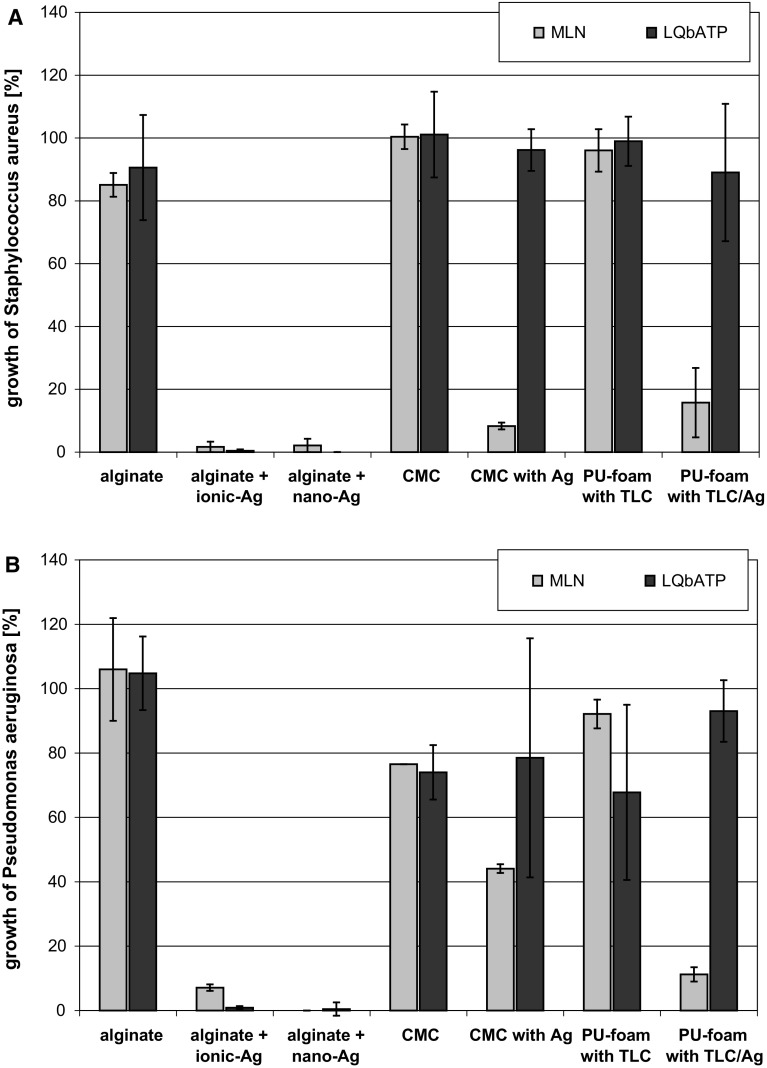
For evaluation of the MLN and LQbATP assay results, data was transformed to read the growth of *S. aureus* (**a**) and *P. aeruginosa* (**b**) in [%] compared to the medium control. It was found that the basic materials alginate, CMC, and PU-foam with TLC did not significantly affect bacterial growth. Extracts of alginate + ionic-Ag and alginate + nano-Ag demonstrated a complete reduction of *S. aureus* and *P. auruginosa*. Differing results were found for the extracts of CMC with Ag^+^ and PU-foam with TLC/Ag^+^. Here, the LQbATP did not show an equal reduction of microbial growth compared to MLN where inhibition of bacterial growth was clearly observable

Using LQbATP, similar results were obtained in regard to the basic materials that did not significantly affect bacterial growth (Fig. 3). Moreover, extracts of alginate + ionic-Ag and alginate + nano-Ag also demonstrated a complete reduction of *S. aureus* and *P. auruginosa*. However, differing results were found for the extracts of CMC with Ag^+^ and PU-foam with TLC/Ag^+^ against both, *S. aureus* (Fig. 3a) and *P. aeruginosa* (Fig. 3b). Here, the LQbATP did not show an equal reduction of microbial growth compared to the MLN where the inhibition of bacterial growth was clearly observable.

### Employing ADT and MLN to evaluate the pH effect on antibacterial activity of silver-dressings

**Fig. 4:**
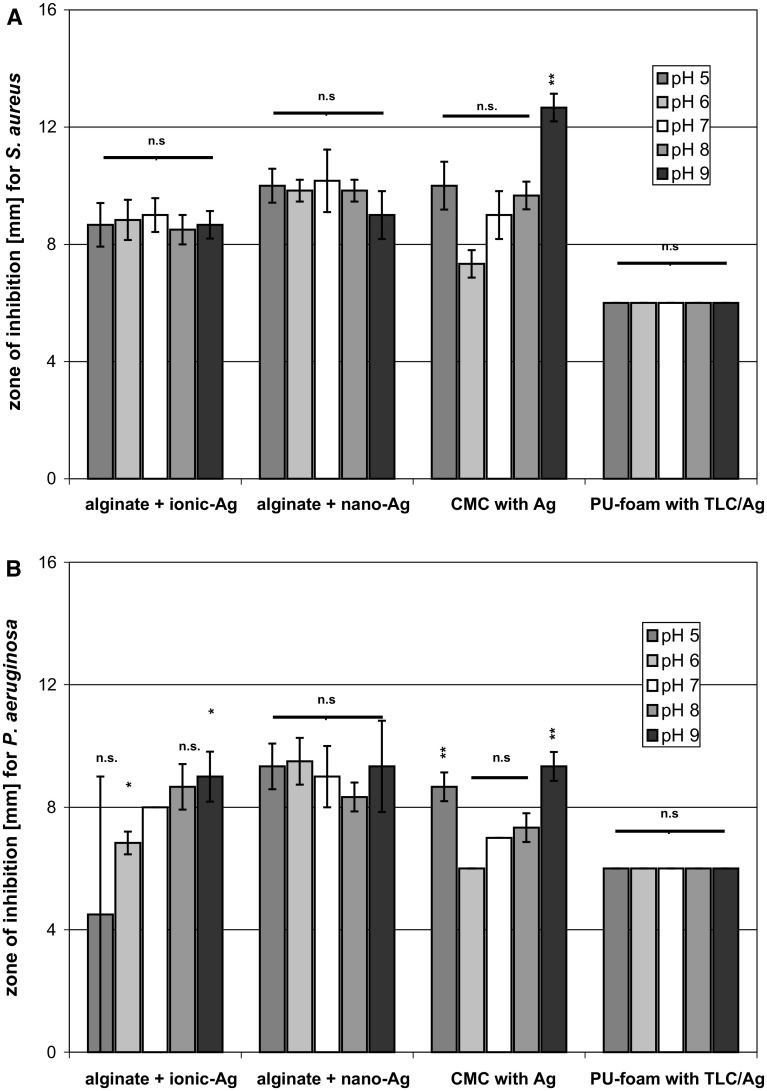
For determination of the pH influence on antibacterial efficacy using the ADT, caso-agar plates with different pH were inoculated with *S. aureus* (**a**) or *P. aeruginosa* (**b**) and incubated with the silver-containing dressings at 37 °C for 24 h. Subsequently, the zone of inhibition (ZOI) was measured

For testing the effect of the pH on antibacterial efficacy in the ADT, caso-agar plates with pH 5.0, 6.0, 7.0, 8.0, and 9.0 were inoculated with *S. aureus* (Fig. 4a) or *P. aeruginosa* (Fig. 4b). Alginate + nano-Ag caused an average ZOI of 10 mm and was equally effective against *S. aureus* and *P. aeruginosa*. No change in the antibacterial effect was further observed for PU-foam with TLC/Ag^+^ at the different pH. In contrast, CMC with Ag^+^ exhibited an increased ZOI against *S. aureus* at pH 9.0 and against *P. aeruginosa* at both, pH 5.0 and pH 9.0. Interestingly, alginate + ionic-Ag did not reveal a pH-dependency of the ZOI formation in the test against *S. aureus*. However, against *P. aeruginosa* a significant increase of the antibacterial effect from pH 5.0 to pH 9.0 was observed for alginate + ionic-Ag.

**Fig. 5:**
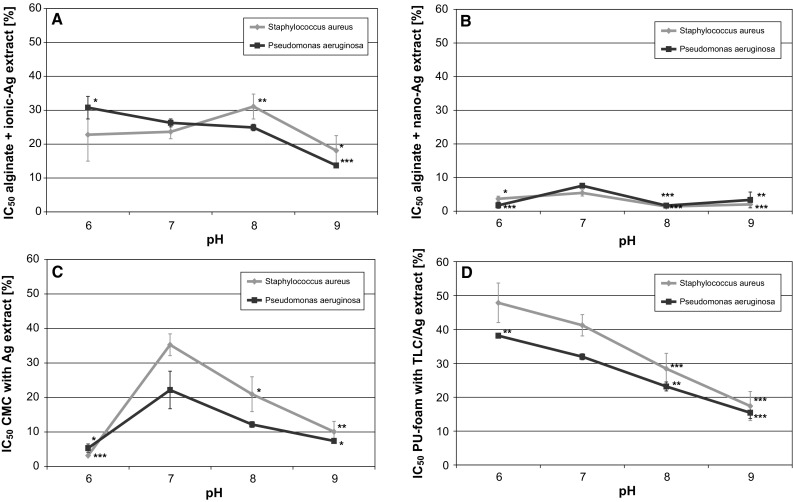
The specific IC_50_, the half maximum inhibitory concentrations, were calculated from the dose–response curves recorded at different pH for the extracts of alginate + ionic-Ag (**a** ), alginate + nano-Ag (**b**), CMC with Ag^+^ (**c**) and PU-foam with TLC/Ag^+^ (**d**) using MLN

It was found that low pH (5.0) already effectively inhibited microbial growth in solution. While no significant difference in the growth of *S. aureus* and *P. aeruginosa* was observed at pH 6.0–9.0, their progeny at pH 5.0 was found to be reduced to less than 10 % of the control at pH 7.0 (own unpublished results). Hence, MLN tests were only performed at a pH range of 6.0–9.0. It could be shown that the silver-dressing extracts possess a pH-dependent antimicrobial activity. For instance, IC_50_ values of the extracts of PU-foam with TLC/Ag^+^ significantly decreased from pH 6.0 to pH 9.0 against both, *S. aureus* and *P. aeruginosa* (Fig. 5d). Alginate + ionic-Ag also exhibited an increase in antibacterial activity for *P. aeruginosa* while IC_50_ values for *S. aureus* slightly increased from pH 6.0 to pH 8.0 and then again dropped at pH 9.0 (Fig. 5a). In addition, alginate + nano-Ag (Fig. 5b) and CMC with Ag^+^ (Fig. 5c) showed similar effects against *S. aureus* and *P. aeruginosa* demonstrating a reduction in IC_50_ values with increasing pH from 7.0 to 9.0. In both cases, a significantly higher antibacterial activity of the extracts was observed at pH 6.0.

## Discussion

In vitro tests allow the direct comparison of antimicrobial effects of wound dressings. However, the method selected will influence the results obtained and therefore, the evaluation of the antimicrobial activity of a dressing. Here, the antimicrobial activity of silver-containing dressings was compared using standard tests like the agar diffusion test (ADT) and the challenge tests JIS L 1902 and AATCC 100, as well as extraction-based methods such as microplate-laser nephelometry (MLN) and luminometric quantification of bacterial ATP (LQbATP).

In general, all silver-containing dressings exhibited antimicrobial activity in all in vitro tests used. The results correlated considerably well, for instance, a strong antimicrobial effect was determined for alginate + ionic Ag and alginate + nano-Ag by ADT, JIS L 1902, AATCC 100, MLN as well as LQbATP The PU-foam with TLC/Ag^+^ inhibited bacterial growth only directly under the sample and no ZOI was formed in the ADT. In accordance, MLN showed that growth of *S. aureus* and *P. aeruginosa* was less affected by the extract of PU-foam with TLC/Ag^+^ compared to the preparations from alginate + ionic-Ag and alginate + nano-Ag. These results indicate that the active agent silver is released in lower amounts by the PU-foam with TLC dressing because it either is present in lesser concentrations or is more closely bound in this material compared to the other dressings with alginate and carboxymethylcellulose. The higher activity of CMC with Ag^+^ against *S. aureus* compared to *P. aeruginosa* in the ADT translated into a stronger effect of the extract on the growth of the gram-positive germ by contrast with the gram-negative test species in the MLN. Minor antibacterial effects of CMC with Ag+ compared to the silver-containing alginates might again be explained by lower amounts of silver ions present as well as effects of the basic materials themselves conveying antibacterial activity. Moreover, delivery of the ionic-Ag in the alginate dressing might be miore readily and the effect of nano-Ag more sustained. Differing results were observed using LQbATP to determine the antimicrobial efficacy of extracts of CMC with Ag^+^ and PU-foam with TLC/Ag^+^ compared to MLN. Here, the LQbATP did not show any or only a moderate reduction of microbial growth while MLN clearly indicated the inhibition of bacterial growth. It is most likely that the dressings affect the LQbATP assay by rendering the pH. This could have an influence on the test outcome as the determination of viable bacteria in the assay depends on cell lysis which is as is known pH-sensitive.

As expected, neither the ADT nor the extraction-based methods demonstrated antimicrobial activity of the silver-free dressings. However, antimicrobial effects of the basic materials alginate, carboxymethylcellulose (CMC) and polyurethane with TLC (PU-foam with TLC) were found in the challenge tests. Both, JIS L 1902 and AATCC 100 revealed a strong antimicrobial activity with log-reduction values >3 (JIS L 1902) or a reduction of germs >90 % (AATCC 100) for alginate and CMC against both *S. aureus* and *P. aeruginosa*. The PU-foam with TLC achieved a slight antimicrobial activity against *S. aureus* and a strong effect on *P. aeruginosa* according to JIS L 1902. Solely in the AATCC 100 test, the PU-foam with TLC did not succeed in decreasing the numbers of *P. aeruginosa*. However, at least a significant antimicrobial activity was obtained by evaluation of the same results according to JIS L 1902. This is due to the fact that JIS L 1902 rates the total antibacterial activity where the reduction of micro-organism growth by the test sample is evaluated in comparison to a polyester growth control after the respective incubation period according to Eq. 1. In contrast, the AATCC 100 rates the reduction of germs by the sample after the incubation period to the number of micro-organisms at starting point (0 h) as stated by Eq. 2. While the PU-foam with TLC was able to impede *P. aeruginosa* progeny, bacteria numbers still increased over the incubation period. Hence, no antibacterial effect is found according to the AATCC 100. However, as numbers of germs in the PU-foam with TLC samples are lower than that of the polyester growth control, a significant antimicrobial activity against *P. aeruginosa* is issued according to the JIS L 1902.

That the basic materials alginate and CMC exert an antimicrobial influence at all in the challenge tests is due to their gelling properties. Alginate and CMC fibers absorb water and swell, the spaces between the fibers are closed and bacteria are trapped [20, 27, 28]. In accordance, the dressings demonstrated a significantly higher effect on *P. aeruginosa* compared to *S. aureus*. This is due to the fact that gram-negative species are far more susceptible to the water deprivation caused by the fluid up-take than the gram-positive *S. aureus* (Prof. Kramer, personal communication). Although the PU-foam with TLC does not produce a gel, it does provide a high fluid absorption capacity and might immobilize bacteria inside its matrix. This could be supported by interactions between the TLC-components of the PU-foam with the cell wall of the bacteria, which would also explain the differences observed for the gram-positive *S. aureus* and the gram-negative *P. aeruginosa*.

This study clearly demonstrates that method features have to be taken into account for selection of a specific test and interpretation of the results. As bacteria in challenge tests come into direct contact with the material, not only items containing an antimicrobial agent, such as silver, might exert an effect but also samples with high absorptive capacities that wield the ability to immobilize micro-organisms. In contrast, the ADT registers only antimicrobial materials that contain a diffusible agent. It is a simple test that can be used to compare different samples under consistent conditions but does not yield a quantitative evaluation. Here, extraction-based methods such as MLN and LQbATP are advantageous as they allow measurement of microbial growth and quantification of viable micro-organisms, respectively. However, they will also only assess a material as antimicrobially effective if it contains an extractable antimicrobial agent. To distinguish whether a material may convey an active effect or a passive mechanism, it is recommended to choose tests from both groups.

Conversely, if a specific question is raised, e.g. how the wound pH affects the antibacterial efficacy of antimicrobial-containing dressings, tests that address passive mechanisms are not needed while experiments that assess both, the influence on diffusion capacity and the extraction capability should be included. Hence, ADT and MLN were elected to investigate the effect of pH on antimicrobial activity of silver-containing dressings. In vitro studies showed that wound dressings can have significant effects on the pH [26]. In accordance, alginate + ionic-Ag and CMC with Ag^+^ led to a more acidic environment in vitro while alginate + nano-Ag stabilzed the pH around 8.0 (Table 2). PU-foam with TLC/Ag^+^ had no effect on the pH. Similarly, the question has to be raised, if wound pH vice versa affects the activity and efficacy of antimicrobial dressings. Recent studies relating to the influence of the pH on the performance of antiseptics revealed that bactericidal activity of chlorhexidine and octenidine was mainly pH-independent in the pH range from 5.0 to 9.0 while a most pronounced influence was observed for polihexanide and PVP-iodine (own unpublished results). In the ADT, pH-independent formation of ZOI was obtained for alginate + ionic Ag, alginate + nano-Ag, and PU-foam with TLC/Ag^+^ against *S. aureus* while a slight increase of the effect of CMC with Ag^+^ was observed at pH 9.0. This is in accordance with a study by Braunwarth et al. which showed that silver-containing dressings possess similar bacteriostatic effects over a pH range of 5.5–9.0 [29]. However, against *P. aeruginosa* only alginate + nano-Ag and PU-foam with TLC/Ag^+^ exerted consistent effects at the different pH while CMC with Ag^+^ demonstrated formation of larger ZOI at pH 5.0 and 9.0 and alginate + ionic-Ag exhibited a significant increase of ZOI formation from pH 5.0 to 9.0. Comparable effects were observed in the MLN measurements at different pH for alginate + ionic-Ag and CMC with Ag^+^ against both, *P. aeruginosa* and *S. aureus*. IC_50_ values for the alginate + nano-Ag extracts were also decreased at pH 6.0, 8.0 and 9.0 compared to pH 7.0. Most notable was the effect by CMC with Ag^+^ at pH 6 which is most likely due to the acidification of the solution by the dressing itself (Table 2). It was found that planktonic bacteria in solution are quite sensitive to environmental changes in the pH [30], for instance, neither *S. aureus* nor *P. aeruginosa* could be grown in medium at pH 5.0 for MLN measurements while no effect on these micro-organisms was found growing them on agar with pH 5.0 for the ADT (own unpublished results). Moreover, a more alkaline environment might also increase the susceptibility of bacteria to antimicrobial agents [30]. Hence, it can be expected that dressings’ effects on pH and release of silver ions act synergistically for antimicrobial efficacy.

## Conclusions

In conclusion, it could be shown that the method selected for determination of antimicrobial effects of wound dressings will influence the results obtained and therefore, the evaluation of the antimicrobial efficacy. Hence, this has to be taken into account for selection of a specific test and the interpretation of data. The agar diffusion test ADT is a simple test that can be used to register antimicrobial materials containing diffusible agents, but it does not yield a quantitative evaluation. While challenge tests like JIS L 1902 or AATCC 100 do provide a quantitative result in form of log-reduction values or the decrease of viable germs in [%], they also encompass the effect of materials with high absorptive capacities that wield the ability to immobilize micro-organisms. Here, the new extraction-based methods MLN (microplate-laser nephelometry) and LQbATP (luminometric quantification of bacterial ATP) can close the gap, as they only assess a material as antimicrobially effective if it contains an extractable antimicrobial agent and supply quantification of microbial growth and viable micro-organisms, respectively. In general, it can be recommended to choose several tests to distinguish whether a material may convey an active effect or a passive mechanism. Moreover, to be informative for clinical application, further tests should be performed in the presence of wound exudate and/or necrotic tissue as these may profoundly affect the antimicrobial activity in vivo.

Moreover, it is of interest to investigate the influence of the pH on the performance of antimicrobial wound-dressings as chronic wounds most commonly have a pH range of 6.5–8.5 [24, 25]. This shift towards higher pH values in chronic wounds compared to acute wounds is called ‘alkaline shift’ and is thought to be due to both, tissue necrosis and the presence of microorganisms. Here, ADT and MLN were used to investigate the influence of pH on antimicrobial activity of selected silver-containing dressings. The results obtained show that the release of silver and hence its antimicrobial efficacy is partially pH-dependent. In addition, the dressings themselves may affect the pH. From the results it can further be speculated that dressings’ effects on pH and release of silver ions act synergistically for antimicrobial efficacy. Hence, it can be expected that the silver-containing dressings alginate + ionic-Ag and CMC with Ag^+^ aid wound healing by antimicrobial effects of silver as well as the establishment of a low physiological pH.

## Abbreviations

AATCC: American Association of Textile Chemists and Colorists
ADT: Agar diffusion test
CMC: Carboxymethylcellulose
JIS: Japanese Industrial Standard
LQbATP: Luminometric quantification of bacterial ATP
MLN: Microplate laser nephelometry
TLC: Lipidocolloid technology matrix
PU: Polyurethane
RNU: Relative nephelometric units
ZOI: Zone of inhibition
